# Mimicking Tumor Hypoxia in Non-Small Cell Lung Cancer Employing Three-Dimensional In Vitro Models

**DOI:** 10.3390/cells10010141

**Published:** 2021-01-12

**Authors:** Iwona Ziółkowska-Suchanek

**Affiliations:** Institute of Human Genetics, Polish Academy of Sciences, Strzeszyńska 32, 60-479 Poznań, Poland; iwona.ziolkowska@igcz.poznan.pl; Tel.: +48-(61)-6579219; Fax: +48-61-823323

**Keywords:** lung cancer, hypoxia, tumor microenvironment, three-dimensional, in vitro models

## Abstract

Hypoxia is the most common microenvironment feature of lung cancer tumors, which affects cancer progression, metastasis and metabolism. Oxygen induces both proteomic and genomic changes within tumor cells, which cause many alternations in the tumor microenvironment (TME). This review defines current knowledge in the field of tumor hypoxia in non-small cell lung cancer (NSCLC), including biology, biomarkers, in vitro and in vivo studies and also hypoxia imaging and detection. While classic two-dimensional (2D) in vitro research models reveal some hypoxia dependent manifestations, three-dimensional (3D) cell culture models more accurately replicate the hypoxic TME. In this study, a systematic review of the current NSCLC 3D models that have been able to mimic the hypoxic TME is presented. The multicellular tumor spheroid, organoids, scaffolds, microfluidic devices and 3D bioprinting currently being utilized in NSCLC hypoxia studies are reviewed. Additionally, the utilization of 3D in vitro models for exploring biological and therapeutic parameters in the future is described.

## 1. Introduction

Worldwide, lung cancer remains the most commonly diagnosed cancer and the greatest cause of cancer-related death. Globally, according to the latest GLOBOCAN 2018 estimates, lung cancer is the most often diagnosed malignancy (2.1 million new cases) with an age-standardized incidence rate of 22.5 per 100,000 person years worldwide in 2018. In both sexes combined, lung cancer is the most commonly diagnosed cancer (11.6% of the total cases) and the leading cause of cancer death (18.4% of the total cancer deaths) [1]. The low survival rate in lung cancer patients is related to the disease at diagnosis [2]. Although, nowadays, there are many new approaches for lung cancer therapies, the 5-year survival rate is still as low as 5–15% [3]. Adenocarcinoma is one of the three major subtypes of non-small cell lung cancer (NSCLC) and is the most common histologic subtype of lung cancer in men and women [4]. The anticancer treatments are based on chemotherapy, radiation therapy and targeted therapy. The major problem is clinical resistance, in which hypoxia is one of the key components. Oxygen deprivation results in gene expression changes and subsequent proteomic changes that have many important effects on various cellular and physiological functions and lead to therapy resistance [5]. Moreover, oxygen deprivation observed among other respiratory diseases such as severe obstructive sleep apnea (OSA) and chronic obstructive pulmonary disease (COPD) may play a role in the initiation and progression of lung cancer [6].

**Figure 1:**
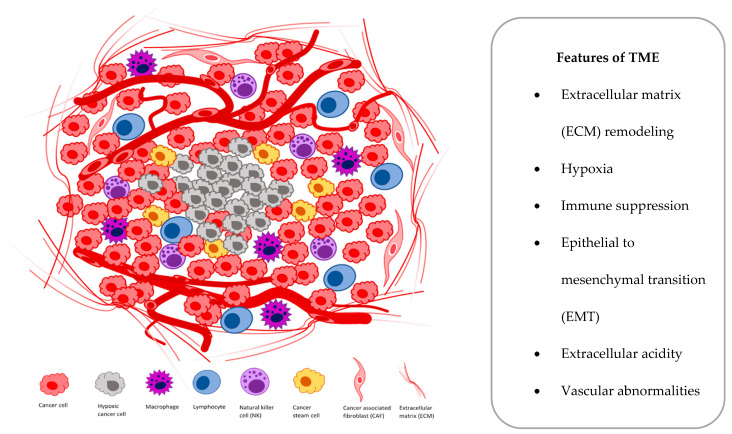
Main components and features of the tumor microenvironment (TME).

At the cellular level, hypoxia is a significant tumor feature, which induces both proteomic and genomic changes within tumor cells, which cause many changes in the tumor microenvironment (TME). The TME is composed of different cells that are programmed to promote initiation, progression and metastasis of lung cancer and various secreted factors and extracellular matrix (ECM), which provides support to the surrounding cells (Figure 1).

The focus of this review is to summarize the data in the field of NSCLC tumor hypoxia, including biology, biomarkers, in vitro and in vivo studies and hypoxia imaging and detection. Moreover, a systematic review of some of the current NSCLC 3D models that have been able to mimic the hypoxic TME is presented. Additionally, the utilization of 3D in vitro models for exploring biological and therapeutic parameters in the future is described.

## 2. Lung Tumor Hypoxia

### 2.1. Biology

Hypoxia is a typical microenvironment feature in nearly all solid tumors. The rapid and uncontrolled proliferation of tumors limits the availability of oxygen and causes insufficient blood supply. Low oxygen level is due to irregularities in tumor vascularization or distance from supporting blood vessels. The diffusion limit for oxygen is ~100–200 μm, which means that for adequate oxygenation cells must be within this radius, which is complicated during tumor growth [7]. The median oxygen level in majority of malignancies is about 10 mmHg, while the normal tissues have oxygen pressure between 40 and 60 mmHg [8]. The severity of hypoxia varies between tumor types and the oxygen level in hypoxic tumor tissues is poorer than the oxygenation of the respective normal tissues (most tumors exhibiting median oxygen levels <2%). This is partially dependent on the tissue of origin [8]. Oxygen concentration in normal human lung tissue is approximately 5.6% O_2_, whereas in NSCLC, tumor is between 1.9–2.2% [9]. Hypoxia generates intratumoral oxygen gradients, contributing to the plasticity and heterogeneity of tumors. Three major forms of hypoxia occur in solid tumors: acute, chronic and intermittent or cycling hypoxia [10]. Acute hypoxia occurs in areas adjacent to blood supply due to transient vessel occlusion. In chronic hypoxia, the blood vessel is patent, however the diffusion of oxygen is limited [11]. The third case was characterized by cyclic periods of hypoxia and reoxygenation [10,12]. E. Marhuenda et al. showed that intermitted and sustained hypoxia profiles differentially stimulated lung cancer cell proliferation [6]. However, the common feature of all forms of hypoxia is correlated with more aggressive tumor phenotype and worse patient outcome [13,14].

### 2.2. Consequences at Cellular and Molecular Level

Hypoxia induces both proteomic and genomic changes within tumor cells, which cause many alternations in the TME. These changes may initiate cell cycle arrest, differentiation, necrosis and apoptosis [15]. Opposite, some changes may stimulate tumor growth, invasion, metastasis, activate and promote angiogenesis, anaerobic metabolism and other processes facilitating cancer cells to survive or escape their low oxygen environment [13]. At a molecular level, the adaptation of tumor cells to hypoxia is regulated largely by the hypoxia-inducible factor (HIF), a transcription factor, which accumulates in response to decreased cellular oxygen levels. Three human HIF family members have been identified: HIF-1, HIF-2 and HIF-3 [15]. All forms bind to hypoxia-response element (HRE) consensus sites and activate different transcriptional responses, which are relevant for hypoxia signaling and oxygen homeostasis in cells [3,16,17].

**Figure 2:**
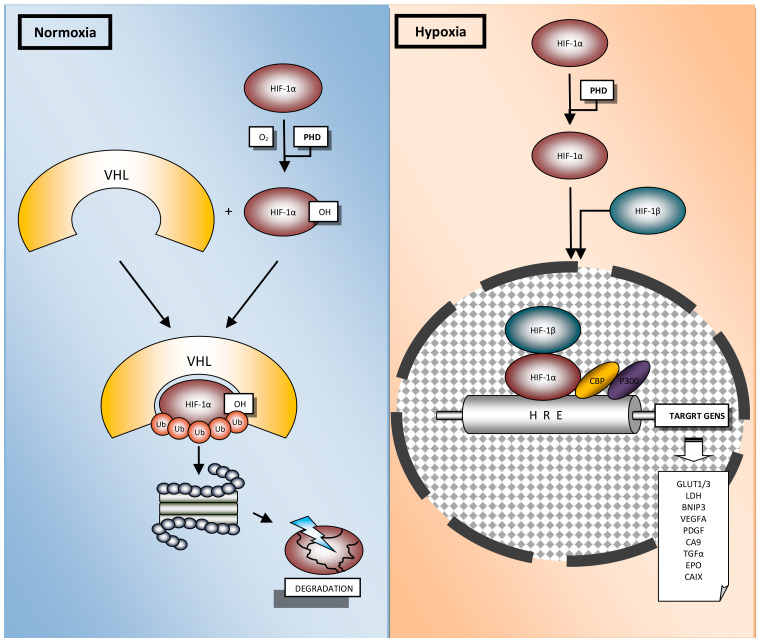
Regulation of HIF-1α in physiological oxygen concentration (normoxia) and hypoxia. In normoxia, HIF-1α is rapidly degraded via ubiquitin-mediated process. The hydroxylation of HIF-1α by prolyl hydroxylase domain proteins (PHD), with use of oxygen, leads to interaction with Von Hippel–Lindau (VHL), addition of ubiquitin (Ub) and HIF-1α proteasomal degradation. Under oxygen deprivation, HIF-1α is not targeted for degradation. Hypoxia leads to HIF-1α accumulation in the cytoplasm, whence it is translocated to the nucleus where it binds with HIF-1β. α and β subunits form the heterodimer HIF-1, which joins to the hypoxia response element (HRE) and recruits co-activators (CBP, p300) at the HRE to induce the transcriptional activity of target genes.

The main player of hypoxia induced processes is HIF-1, a heterodimeric transcriptional factor composed of the highly regulated HIF-1α subunit and the constitutively expressed HIF-1β (ARNT, aryl-hydrocarbon receptor nuclear transporter) (Figure 2) [18,19].

**Figure 3:**
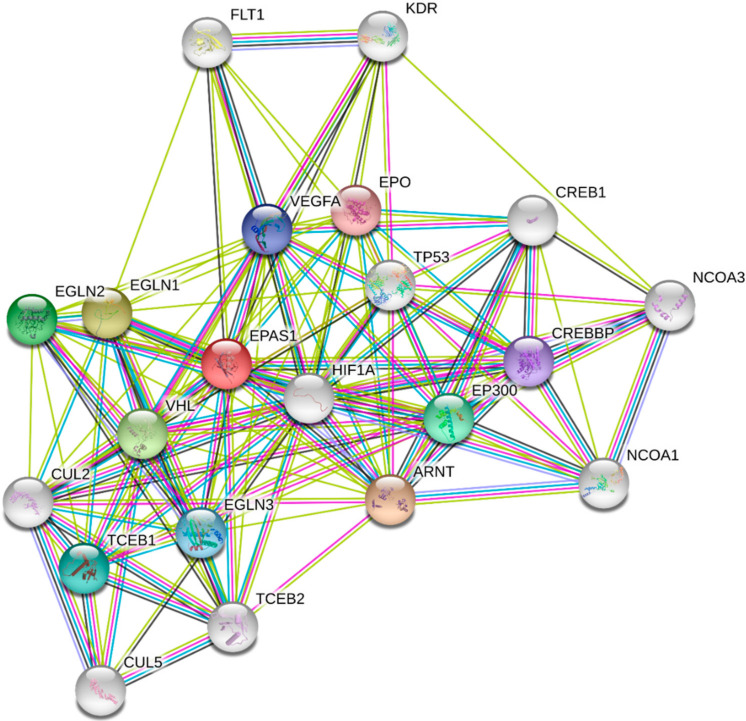
The protein–protein interaction network of HIF-1α obtained from String v11 database [29]. The colored lines of the edges represent the existence of different types of evidence used in predicting the associations (green: neighborhood evidence; blue: co-occurrence; purple: experimental evidence; light blue: database evidence; black: co-expression evidence). The HIF-1α interacting proteins are (in alphabetical order): ARNT: aryl hydrocarbon receptor nuclear translocator; CREB1: cyclic AMP-responsive element-binding protein 1; CREBBP: CREB-binding protein; CUL2: cullin2; CUL5: vasopressin-activated calcium-mobilizing receptor 1; EPAS: endothelial PAS domain protein 1, HIF-2α; EGLN1: Egl nine homolog 1; EGLN2: Egl nine homolog 2; EGLN3: Egl nine homolog 3; EPO: erythropoietin; EP300: histone acetyltransferase p300; FLT1: vascular endothelial growth factor receptor 1; KDR: vascular endothelial growth factor receptor 2; NCOA1: nuclear receptor coactivator 1; NCOA3: nuclear receptor coactivator 3; TCEB1: elongin-C; TCEB2: elongin-B; TP53: cellular tumor antigen p53; VEGFA: vascular endothelial growth factor; VHL: Von Hippel–Lindau disease tumor suppressor.

HIF-1α is a pivotal hypoxia transcription factor, which regulates expression of many genes. Degradation of HIF-1α under normoxic conditions is a ubiquitin-mediated process, activated by the tumor suppressor protein pVHL (Von Hippel–Lindau protein) [20,21]. In normoxia, HIF-1α is rapidly degraded and its extended lifetime is 30 min. [22]. Oxygen deprivation leads to HIF-1α accumulation in the cytoplasm, whence it is translocated to the nucleus [23]. Afterwards, it interacts with the HIF-1β subunit to form the heterodimer HIF-1, which binds to the consensus sequence 5′-(A/G) CGTG-3′ HREs to induce the transcriptional activity of target genes [24,25]. It is known that the HIF-1 heterodimer transcriptionally regulates more than a thousand genes [26,27]. This modulation includes genes involved in the uptake and metabolism of glucose (*GLUT1, LDH*), apoptosis (*BNIP3*), angiogenesis (*VEGFA, PDGF*), control of extracellular pH (*CA9*), mitogenesis (*TGFα*) and erythropoiesis (*EPO*) (Figure 2) [28]. Additional proteins, which are involved in the HIF-1α protein–protein interaction network, presented in Figure 3, were derived from String v11 database [29].

## 3. Role of HIFs in Lung Cancer

### 3.1. In Vitro Studies

**Figure 4:**
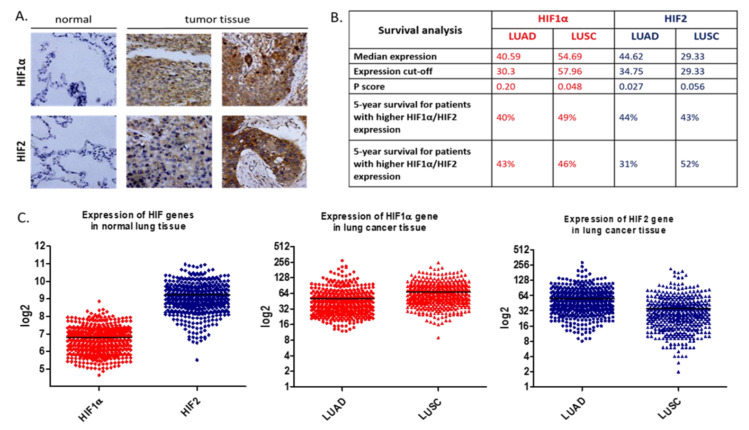
(**A**)**.** HIF-1α and HIF-2α expression in non-small cell lung cancer (NSCLC) tumors. (**B**)**.** Survival analysis of lung adenocarcinoma (LUAD, *n* = 500) and lung squamous cell carcinoma (LUSC, *n* = 494) patients. Median expression refers to the median FPKM value calculated based on the gene expression (FPKM) data from all patients in this dataset. Expression cut-off: based on the FPKM value of each gene, patients were classified into two groups, and association between survival and gene expression (FPKM) was examined. The best expression cut-off refers the FPKM value that yields maximal difference with regard to survival between the two groups at the lowest log-rank P-value. P score: Log-rank P value for Kaplan–Meier plot showing results from analysis of correlation between mRNA expression level and patient survival. Five-year survival for patients with higher or lower expression than the expression cut-off. (**C**)**.** Expression of HIF-1α and HIF-2α in lung adenocarcinoma (LUAD) and lung squamous cell carcinoma (LUSC) tumor tissue derived from patients and healthy controls. All data presented in Figure 3 were collected from The Human Protein Atlas version 20.0 database [33,34].

The positive association between hypoxia and tumorigenesis is well known, and accordingly, HIFs protein expression is correlated with more aggressive and radiation-resistant tumor cells [30]. Previous studies have shown that the level of HIF-1α and HIF-2α is increased in NSCLC and both are associated with poor patient prognosis [31,32]. HIF-1α and HIF-2α expression in NSCLC tumors is summarized in Figure 4 [33,34]. Studies on early stage resectable NSCLC revealed that HIF-1α and HIF-2α proteins were found in 62 and 50% of samples, respectively. What is more, elevated HIF-2α expression correlated with an increased density of microvessels, which were VEGF positive in immunohistochemical staining [31]. The hypoxia-induced *VEGF* mRNA was also detected in highly metastatic lung cancer cells [35,36].

For some of the clinical and in vitro studies, more prominent relevance of HIF-2α subunit compared to HIF-1α as an unfavorable prognosis biomarker in NSCLC was found. The meta-analysis revealed strong significant negative associations between HIF-2α expression and overall survival, disease-free survival, disease-specific survival, metastasis-free survival and progression-free survival [37]. HIF-2α expression but not HIF-1α was related to poor outcome and tumor size, lymph node metastasis, tumor stage and histology [38]. Moreover, HIF-2α was highly expressed in cancer stem cells, which have been associated with a radioresistant phenotype in lung cancer [39]. J. Bertout et al. demonstrated that inhibition of HIF-2α expression augmented p53 activity, increased apoptosis and reduced clonogenic survival of irradiated and non-irradiated A549 human lung adenocarcinoma cells [40].

The role of HIF-1/2α in radiation sensitivity of NSCLC was also investigated with the use of CRISPR gene-editing of H1299 cells lacking HIF-1α, HIF-2α or both. Among HIF-α isoform-deficient cells the authors identified a strong radiosensitizing effect of HIF-1α, but not of HIF-2α, which was associated with a decreased extracellular pH and reduced glycolysis [41].

### 3.2. In Vivo Studies

In vivo models were commonly used to assess the role of HIFs in cellular processes and cancer development. Heterozygous Hif-1α^+/−^ mice exposed to chronic hypoxia (10% O_2_, one to six weeks) developed ventricular hypertrophy, pulmonary hypertension and pulmonary vascular remodeling compared with wild-type littermates [42]. It was also reported that HIF-1-mediated alterations are crucial in hypoxia-induced autophagy. Experiments on Hif1a^-/-^ knockout mouse embryo fibroblasts revealed that mitochondrial autophagy is an adaptive metabolic response that promotes the survival of cells under conditions of prolonged hypoxia. This process requires the HIF-1-dependent induction of BNIP3 (BCL2 interacting protein 3) [43].

Nevertheless, there are some discrepancies in the results obtained from transgenic mouse/rat experiments in lung cancer research. In mice injected with HIF-1α depleted A549 cells, impaired tumor vascularization and increased necrotic area was observed. However, the reduction in tumor cell proliferation and tumor growth was not present [44]. Another study on xenograft models showed that deletion of HIF-1α in the mammary epithelium resulted in decreased pulmonary metastasis [45].

Effectiveness against lung tumor growth was also observed in vivo after treatment with HIF-1α inhibitors. In an orthotopic mouse model of human NSCLC, treatment with a small molecule inhibitor of HIF-1α, PX-478, significantly reduced the median primary lung tumor volume [46]. The antitumor activity against NSCLC has also been demonstrated for another inhibitor, SCH66336. J. Y. Han et al. indicated that SCH66336 inhibits angiogenic activities of NSCLC cells by decreasing hypoxia and also reduction in VEGF production probably by the interaction between HIF-1α and Hsp90, resulting in the proteasomal degradation of HIF-1α [47].

In recent years, there has been growing interest in the relationship of HIF-2 with progression and prognosis in patients with NSCLC. In vivo studies on mice that conditionally expressed HIF-2α and a mutant form of *Kras* (Kras^G12D^) revealed that tumors demonstrated features of epithelial-mesenchymal transition (EMT) and exhibited increased angiogenesis associated with mobilization of circulating endothelial progenitor (CEP) cells [48]. Another study obtained from Kras^G12D^-driven murine NSCLC model showed that, opposite to Hif-1α, Hif-2α deletion resulted in increased tumor burden that correlated with reduced expression of the candidate tumor suppressor gene *Scgb3a1* (HIN-1, Secretoglobin Family 3A Member 1) [49].

Potential HIF-1α inhibitors were most commonly investigated compared to inhibitors against HIF-2 [50]. In NSCLC cells, a novel lead structure, BAY 87-2243, identified through high-throughput screenings, was tested. This inhibitor suppressed the accumulation of HIF-1α and HIF-2α proteins in cancer cells by inhibiting mitochondrial complex I activity, but it did not pass the phase I clinical trials [51].

## 4. Hypoxia Assessment in NSCLC

There are several methods for detecting tumor hypoxia, but because of heterogeneity in tumor oxygen levels, there is no ideal hypoxia detection strategy. In lung cancer only, three methods have been employed to assess hypoxia: (a) measurement of partial oxygen pressure with needle electrodes, (b) imaging hypoxia and tumor vasculature and (c) detection of hypoxia-induced proteins in tumor/blood [52].

### 4.1. Oxygen Electrodes

Direct partial pressure of oxygen (pO_2_) measurements was made by oxygen electrodes in patients undergoing surgery for early resectable NSCLC [9]. Using electrodes, more than a hundred measurements are generated over the accessible areas of the lesion. However, the construction of 3D oxygen maps makes using electrodes difficult, despite a spatial resolution of 50–100 cells, preventing electrode-based therapy planning. Additionally, the probe does not discriminate between viable and necrotic tissue; thus, it overestimates hypoxia when necrotic areas are sampled [53]. As the technique is suitable only for accessible tumors and may lead to severe complications such as pneumothorax in case of NSCLC tumors, it is not widely used in clinical practice [54].

### 4.2. Imaging Hypoxia

In clinical routine, there is a need for noninvasive techniques that can reliably detect hypoxia. Patient stratification by hypoxia status may be very useful in personalized medicine connected with hypoxia targeting strategies [55]. Generally, the most studied noninvasive techniques to visualize hypoxia make use of positron emission tomography (PET) tracers based on 2-nitroimidazoles labeled with fluorine-18, e.g., ^18^F-MISO, ^18^F-FAZA and ^18^F-HX4 [56]. The absence of oxygen leads to the reduction in nitroimidazoles, which cannot be reversed, and the reactive product gets trapped in the hypoxic cell [55]. Fractional hypoxic volume, defined by ^18^F-MISO PET, was reduced from 58 to 22% after radiotherapy of seven patients with locally advanced NSCLC [50]. ^18^F-FAZA PET allowed to detect heterogeneous distributions of hypoxic sub-volumes on visual analysis. The potential clinical value of the specific hypoxia tracer ^18^F-FAZA over commonly used ^18^F-FDG in the treatment of patients with stage III-IV NSCLC was estimated. Therefore, ^18^F-FAZA might be considered as a tool for guiding dose escalation to the hypoxic fraction of the tumor [57]. ^18^F-FETNIM uptake was higher in tumors compared to normal tissue. Moreover, FETNIM uptake was correlated with a worse outcome and with the expression of HIF-1α, GLUT-1 and VEGF, all upregulated under hypoxic conditions [58]. The feasibility of other PET tracer [^60^Cu]-ATSM in patients with NSCLC was confirmed by F. Dehdashti et al. The tumor uptake of ^60^Cu-ATSM revealed clinically relevant information about tumor oxygenation that is predictive of tumor response to therapy [59].

Perfusion- and diffusion-based approaches, such as computed tomography (CT)-based perfusion techniques, dynamic contrast-enhanced CT (DCE-CT), as well as magnetic resonance imaging-based (MRI-based) modalities, DCE-MRI or diffusion-weighted MRI (DW-MRI) have been proposed for indirect detection of hypoxia state. Additionally, upregulated metabolism that can be visualized by ^18^F-FDG PET has been suggested as another indirect marker of hypoxia. In NSCLC patients, hypoxia levels were assessed with HX4 PET tracer by using FDG PET/CT and dynamic contrast-enhanced CT imaging [55]. This comprehensive modeling approach enabled the classification of the tumors as normoxic or hypoxic and allowed to assess the observed hypoxic volumes for each patient [55]. However, hypoxia PET research is still ongoing. The integration of hypoxia PET in NSCLC hypoxia-targeted therapy trials was slowed because of high imaging costs, limited radiotracer availability and lack of technical validation. For this reason, efforts should focus on validating the most promising hypoxia radiotracers based on technical characteristics, such as ^18^F-FAZA and ^18^F-HX4 [11].

### 4.3. Detection of Hypoxia-Induced Markers

The well-known exogenous hypoxia marker pimonidazole is a 2-nitroimidazole compound, which forms covalent bonds with cellular macromolecules at low oxygen levels. Immunohistochemical observation of pimonidazole can indicate partial pressure of oxygen levels of less than 10 mm Hg [60]. Blood volume (BV) in NSCLC may be a surrogate marker for tumor hypoxia, because BV reflects the proportion of tissue that comprises flowing blood. Negative correlation between BV and pimonidazole staining was observed in operable NSCLC tumors. The authors revealed that tumors with the low functional BV were more hypoxic [61].

Endogenous markers were also used to assess the distribution of hypoxic areas in NSCLC, because they appear to reflect different aspects of hypoxia compared to exogenous markers. Hypoxia influences protein expression largely through HIF-1, which when activated under hypoxic conditions, leads to the transcription of numerous genes that potentially can be used as hypoxia endogenous biomarkers (Figure 2 and Figure 3). The correlation of HIF-1 and/or HIF-2 expression was widely studied among NSCLC [31]. HIF-1α uniquely stimulates the expression of many enzymes such as lactate dehydrogenase 5 (LDH5) [62] and carbonic anhydrase IX (CAIX) [63], apoptosis (BNIP3) [64], angiogenesis (VEGFA, PDGF) [65] and control of extracellular pH (CA9) [66]. However, HIF-2α stimulates transforming growth factor-α (TGF-α) and erythropoietin (EPO) [67]. On the other hand, genes such as glucose transporters (GLUT1, GLUT3) and VEGF-A are commonly upregulated by both HIF1α and HIF2α [28,68].

Besides the aforementioned molecules, another new potential surrogate of hypoxia is osteopontin (OPN), which has been shown to be associated with intratumoral pO_2_. In a pilot study, high pretreatment plasma levels of OPN combined with CAIX and VEGF were additively correlated with prognosis in M0-stage NSCLC patients receiving radical radiotherapy [69].

Recently, newly identified YTHDF1 hypoxia adaptation gene was associated with NSCLC progression. YTHDF1 inhibition suppressed NSCLC cell proliferation, colony formation, xenograft tumor formation and *de novo* lung ADC progression. Moreover, low expression of this gene correlated with a worse clinical outcome by rendering cancer cells resistant to cisplatin treatment [70].

Circulating hypoxia biomarkers were also identified among NSCLC. A number of plasma microRNA (miRs) are induced by hypoxia, with select members (miR-26, miR-107, miR-210) [11]. There is growing evidence of miR-210 involvement in hypoxic cell radioresistance [71], with conflicting evidence on the prognostic impact of tumor expression of miR-210 in NSCLC patients [72]. High serum miR-210 was linked with advanced stage (III–IV) and poor chemotherapy response in NSCLC patients. However, methodological approaches for circulating biomarkers need to be validated and standardized across laboratories [73].

Hypoxia biomarkers are yet to be validated in NSCLC clinical trials. The major difficulty is that biomarkers are predictive in one therapeutic setting (radiotherapy) might not retain this predictive capacity in different settings (chemoradiotherapy) within the same tumor. Additionally, an NSCLC-specific hypoxia gene expression signature is currently under development, with evidence suggesting different prognostic capability according to tumor histology [11].

## 5. Clinical Implications

The assessment of tumor hypoxia is very relevant in clinical practice and will be valuable to radiation oncologists, surgeons and also pharmaceutical companies who are engaged in developing hypoxia-based therapies or treatment strategies. Detectable hypoxia is present at baseline in around 50 to 80% of stage I–IV NSCLC patients according to several positron emission tomography (PET) studies [11]. Hypoxic tumor cells were thrice more resistant to radiation than well-oxygenated ones, thus hypoxia impairs the effectiveness of radiotherapy [53]. Tumor sensitivity to radiation exposure rapidly declines when the local pO_2_ is less than 25–30 mmHg (3.3–3.9%). In hypoxia situations, the cells have increased ability to conduct repairs to the disrupted DNA, which leads to resistant subpopulations of cancer cells [15].

Hypoxia exacerbates the abnormal growth of vascular networks, which consist of vessels with pathological size, inconsistent expansion and hyperpermeability. Under these conditions, the delivery of agents that are beneficial in the treatment of cancer is ineffective. Moreover, hypoxic tumor cells are distant from the blood supply and may be exposed to a lethal dose of a cytotoxic agent. Hypoxic cells are known to be more resistant toward commonly used drugs, e.g., fluorouracil, doxorubicin, bleomycin and platinum-based drugs than normoxic cells [53]. Because hypoxia is also linked to NSCLC therapy resistance, there are several approaches to target hypoxia via increasing hypoxic cell radiation sensitivity (misonidazole, nimorazole), increasing oxygen delivery (carbogen, nicotinamide, efaproxiral), decreasing oxygen consumption (metformin, atovaquone), specific targeting of hypoxic cells using hypoxia-activated cytotoxic prodrugs (tirapazamine, evofosfamide, tarloxotinib bromide), hypoxia molecular target inhibitors (aryl sulfonamides targets, HIF-1α gene products) and various other hypoxia-related mechanisms (nitroglycerin, BKM120) [11].

The main hypoxia player, HIF-1 remains a promising molecular target that could be used for the development of novel cancer therapeutic strategies. Addition of HIF inhibitors to current treatments for lung cancer may prove beneficial in slowing tumor progression and metastasis [74]. An increasing number of agents are constantly being reported that inhibit HIF-1α expression and/or activity: inhibitors of HIF-1α mRNA expression (antisense oligonucleotide EZN-2968) and protein translation (EZN-2208 agent), inhibitors that affect HIF-1α degradation pathway (Hsp90 inhibitors: 17-AAG and 17-DMAG), HIF-1α inhibitors of DNA binding (doxorubicin and daunorubicin) and inhibitors of HIF-1α transcriptional activity (proteasome inhibitor Bortezomib, PS-341) [74]. Some of HIF inhibitors and small molecules targeting HIF have been more recently described, either being tested in early clinical trials or already approved for use in patients.

To date, hypoxia-targeted therapies have not been adopted as part of standard treatment in NSCLC. Promising therapies should be investigated in selected NSCLC patients in combination with radiotherapy, chemotherapy and biological therapies and also synergistic therapeutic combinations, e.g. hypoxia-targeted therapy and immunotherapy [11].

## 6. 2D Model Limitations in Studying Hypoxia Biology

It is worth mentioning that most of hypoxia markers were discovered using 2D cultures, which do not recapitulate the in vivo TME observed in solid tumors. Two-dimensional in vitro models have some major limitations for the study of hypoxia cancer biology (Table 1).

In traditional 2D cell culture, tumor cells form flat monolayer cell culture on plastic dishes and are exposed to hypoxia using hypoxic chambers that maintain hypoxic conditions over a defined concentration of pO_2_. This kind of cell culture cannot reproduce the spatial oxygen gradients and spatial heterogeneity observed in solid tumors in vivo [75]. Studies have shown that the gene expression profiles, as well as the responses to treatment, in the multicellular spheroid 3D models closely reflected a tumor in vivo situation [76]. Comparison of gene/protein expression reveal that metabolic, cell stress–response, structural, signal transduction and cellular transport proteins were expressed at elevated levels in spheroids compared to 2D-cultured cells [77]. Below, the description of commonly used 3D lung tumors models is presented. These models offer the possibility to study not only lung cancer biology in a hypoxia context but may be very useful for anticancer drugs screenings. All 3D models, described in current paper, are summarized in Table 2.

## 7. Multicellular Lung Tumor Spheroids

The multicellular tumor spheroid (MCTS) model is one of the best-established 3D culture methods. MCTS are aggregates of cancer cells grown in suspension or embedded in gels using 3D culture methods. This model partly recapitulates in vivo tumor microenvironments [97]. Larger MCTS (critical size, 400 µm) sustain oxygen and nutrient gradients that often result in the formation of a necrotic core, similar to those in poorly vascularized tumors, which is crucial in hypoxia studies. There are a few methods designed for MCTS formation: instatic suspension, hanging drop method, spinner and rotational bioreactor, magnetic levitation, microfluidic system and gel embedding [98].

Gene expression differences between a hanging-drop 3D NSCLC model and 2D cell cultures were analyzed by G. Gamerith et al. [99]. Nine hundred and seventy-nine genes were altered in A549 and 1106 in Colo699 cells due to 3D cultivation. Specific GSEA analyses revealed changes in immunologic and endothelial cell proliferation pathways, whereas surprisingly hypoxic, EMT and angiogenic pathways were downregulated [99].

Another novel 3D high-throughput screening system was established by K. Arai et al. with the use of NanoCulture Plates that provided a gel-free micro-patterned scaffold [78]. The relation between the degrees of hypoxia and EMT was quantitated by hypoxia levels of the A549 spheroids treated with various concentrations of TGF-β2. It was shown that hypoxia level of the A549 spheroid was declined with TGF-β2 and elevated with TGF-β receptor I inhibitor (SB431542). Spheroid EMT inhibitory activity of SB431542 was calculated from fluorescence intensities of the Hypoxia Probe. This research approach allowed to utilize the 3D high-throughput screening system in a drug screening of EMT-inhibitory small molecule compounds [78].

A. Amann et al. described the development of a novel tri-culture model, using NSCLC cell lines (A549 and Colo699) in combination with a fibroblast cell line (SV80) and two different endothelial cell lines in a hanging drop technology [79]. Upregulation of hypoxia and vimentin, a-smooth muscle actin, α-SMA (ASMA) and downregulation of E-cadherin were observed in co- and tri-cultures compared to monocultures. Oxygen deprivation was analyzed in this tri-culture model by the expression of carbonic anhydrase IX (CAIX) in microtissues extending beyond 250 µm in diameter. At this diameter, hypoxia starts to play a crucial role due to a critical reduction in oxygen diffusion. CAIX was significantly expressed in microtissues that consisted of A549 cancer cells co-cultured with fibroblasts alone or together with endothelial cells [79].

Another study of X. Lou et al. harnessed the power of spheroids to evaluated matrix metalloproteinase-1 (MMP-1) and VEGF as indicators of the interaction between tumor and stromal cells, employed the 3D cell co-culture collagen gel model, containing human lung adenocarcinoma cells (HCC), human lung fibroblast cells (MRC-5) and macrophages [80]. As MMP-1 and VEGF have been clearly linked to tumor invasion and metastasis, the level of both was monitored in cell culture media under hypoxia and/or serum starvation conditions. It revealed that simulating hypoxia and/or serum starvation conditions induced elevated secretion of VEGF in the 3D co-culture model in vitro but not MMP-1. Authors observed different morphology of HCC in the 2D versus the 3D co-culture system. MMP-1 and VEGF were secreted at higher levels in mixed cell models rather than mono-culture groups, which may reflect physiological metastasis mechanisms as the stromal cells, macrophages and fibroblast cells promote this feature in the TME [80].

As MCTS models based on lung cancer cells have been established, there is still a need to develop a method to generate 3D culture derived from NSCLC tumors. The obtained 3D patient-derived tumor spheroids (PDS) from NSCLC should offer the amenability to drug screening and long-term studies. In a study conducted by Z. Zhang et al., three PDS cultures obtained from stage I/II NSCLC were successfully established and had been continuously cultured over 120 days. Moreover, PDS maintained the cytological features and markers of the primary tumors, because many cells within the tumor spheroids were stained positive for Ki67 and thyroid transcription factor-1. The cytotoxicity of cisplatin in PDS spheroids was also investigated and confirmed that an expandable 3D in vitro NSCLC model is useful for drug screening [81]. The use of spheroid cultures enabled the authors to achieve the model, which closely reflects tumor hypoxia in vivo and allows to conduct the experiments in a long time period.

## 8. Organoids

Organoids are more advanced 3D models that are able to mimic the hypoxic TME. The use of organoids is based on the recreation of miniature arrays of hypoxic cell-derived, self-organizing, tissue-specific outcomes that mimic in vivo counterparts [100].

Organoids can be self-organized into desirable tissue phenotypes and mimic the functionality of an organ, while being composed of one or more cell types [100]. Recently, tissue-specific stem cells derived from several adult human lung have been cultured in 3D conditions using hydrogel with collagen or other ECM components. It was shown that basal, secretory and type II cells can be grown in 3D culture, with or without supporting stromal cells, and under these conditions, they give rise to self-organizing structures [101]. In these conditions, cells proliferate and give rise to differentiated progeny that undergo self-organization. This is due to specific organoid culture conditions, which promote stem cell proliferation and differentiation. The advantage of organoids lies in fact that they represent a functional unit that consists of a hierarchy of stem cells and differentiated cells. Moreover, the establishment of long-term expansion airway organoids derived from cystic fibrosis (CF) patients and established from lung cancer resections and metastasis biopsies was achieved by N. Sachs et al. [82].

Another study presented the use of an organoid model for derivation of organoids from primary lung cancer tissues and paired non-neoplastic airway tissues (five subtypes of lung cancer and five normal bronchial organoids) [102]. These organoids were cultured for long-term expansion over 6 months without any change in spherical organoid morphology and maintained proliferation capacity measured by expression of the Ki67 marker. Additionally, in vitro drug sensitivity testing revealed that organoids respond to drugs based on their genomic alterations. Based on this, authors have postulated that this model may be useful for predicting patient-specific drug responses through in vitro patient-specific drug trials [102]. However, because cancer organoids were derived only from epithelial cells, the main disadvantage of this model is the lack of a cancer microenvironment including stromal cells and immune cells. Authors suggest that to investigate the interaction between lung cancer cells and the TME, a co-culture system with immune/stromal cells or xenograft models is necessary. This challenge was taken by H. Nakamura et al. [83], as described below.

The authors demonstrated that podoplanin-positive cancer-associated fibroblasts (CAFs) mixed with NSCLC PC-9 cells allowed the generation of round and steric aggregates (hybrid cancer organoids). The proliferation index of PC-9 cells in hybrid cancer organoids, containing podoplanin-overexpressing CAFs, was significantly higher than that of PC-9 cells in organoids containing control CAFs. This phenomenon was confirmed among surgically resected human tumors in which the proliferation of adenocarcinoma cells was significantly higher in the case of podoplanin (+) CAFs than in the podoplanin (−) CAFs [83]. Taken together, the presented hybrid cancer organoid model can be a useful tool for evaluating the TME and hypoxia studies.

To summarize, the major problem in studying tumor hypoxia using organoids is the limited capacity of oxygen deprivation because of inadequate surface diffusion, which may cause cell death over more extended culture periods [100]. Despite this, organoids can still be useful for targeted treatment examinations.

## 9. D Scaffolds and Hydrogels

Cells in 3D scaffolds exhibit similar responses to chemotherapy and radiotherapy as cells in vivo and may be used as an important tool in cancer biology. Three-dimensional scaffolds provide signaling and physical support to the attached cells. Various types of synthetic and natural polymers have been used for hydrogel preparation depending on their biocompatibility, water absorbing ability and gel strength [89]. Several biopolymers were used to generate porous scaffolds, which include collagen, gelatin, silk, chitosan, Matrigel and alginate [103]. Additionally, the synthetic polymers, such as PAG, PLGA and PLA, were used for 3D structures generation. However, culturing cells in scaffolds may not capture the cell-to-cell interactions present in aggregated tumors such as in spheroids.

In the study of A. Stratmann et al. [84], a combined in vitro and in silico lung tumor model based on a biological tissue scaffold was generated. Two cell lines with (HCC827) or without (A549) an activating mutation of the *EGFR*, exhibiting different sensitivities to the EGFR inhibitor gefitinib were cultured on a small intestinal submucosa. The in silico models of the two different tumor subgroups, with activating *EGFR* mutation or with *Kras* mutation, resulted in two different gefitinib responses, reflected in proliferation and apoptosis status. Furthermore, the application of TGFβ1 induced tumor cell invasion and EMT in 3D model, which was visible in mesenchymal cell morphology and modified expression of fibronectin, E-cadherin, β-catenin and mucin-1. The authors demonstrated that the combined in vitro and in silico model represents a powerful tool for analysis of signaling networks, especially involved in proliferation, apoptosis, invasion and EMT [84].

A microphysiologic 3D lung model was established by E. Wallstabe et al. [85] and resembled architectural and phenotypical features of primary tumor. Three-dimensional tumors were generated from A549 cell line on a biological scaffold with intact basement membrane (BM) on SISmuc platform. The antitumor function of receptor tyrosine kinase-like orphan receptor 1-specific (ROR1-specific) CAR T cells was evaluated. Authors found that ROR1–CAR T cells are able to penetrate and migrate through a tumor mass and confer a potent antitumor effect over a several-day period. The detected antitumor activity of ROR1–CAR T was specific and very potent against A549 lung cancer. This microphysiologic 3D lung model may be involved in preclinical CAR T cell research [85].

In another study, cells from tumor resections of six patients’ NSCLC were cultured on human embryonic stem cell-qualified Matrigel-coated plates [86]. In this in vitro culturing system, two important elements of the tumor microenvironment were mimicked: lung fibroblast-derived extracellular matrix and physiological hypoxia (5% O_2_). The stabilization of HIF1-α was assessed during the experiment. Although Western blot analysis revealed stabilization of HIF1-α in the hypoxia environments (2D and 3D) after 72 hours, there was a considerable decrease in HIF1-α stabilization in the 3D environment compared to the 2D environment. This 3D system allowed isolation and rapid expansion of stromal progenitors from patient lung tumor resections. These progenitor cell populations in the TME-like environment may be anticancer drug targets, which limits their effects on promoting cancer metastasis [86].

Synthetic scaffolds constructed from porous poly(lactic-co-glycolic acid) (PLGA) microparticles were also successfully used as substrates for A549 lung cancer cell culture. Additionally, these tumor models were screened in vitro for their therapeutic efficacies [87]. However, hypoxia status was not verified in the abovementioned study.

Another study highlighted the use of synthetic scaffolds for assessing lung cancer cell adhesion, polarity and morphology. NSCLC cells derived from metastatic pleural fluid (NCI-H460) were cultured on polyester-based composite, the Variotis tissue scaffold. In this 3D model, cells showed enhanced expression of a membrane protein related to hypoxia CAIX, which reflects functional changes and demonstrates exchanges between cancer cells and their environment [88].

In another study sodium alginate–gelatin (SA-GL) hydrogel was used to print NSCLC patient derived xenograft cells and lung CAFs co-cultures. Both cells were mixed with the hydrogel, printed and showed high printability and cell viability [89]. Spheroid size distribution after 15 days was in the diameter range of 50–1100 µm, which may allow generation of a hypoxic core. The cellular crosstalk was confirmed in this model by overexpression of vimentin, α-SMA and loss of E-cadherin, which promotes EMT. Tumor stroma interactions to mimic in vivo tumor microenvironments were provided in this study.

In a subsequent study, the 3D tissue-like construct was also used to evaluate the metabolic response of lung cancer cells to ionizing radiation [90]. Cells-in-Gels-in-Paper (CiGiP) were prepared by stacking multiple sheets of paper containing cell-embedded hydrogels and efficiently generated a gradient of oxygen and nutrients that decreased monotonically in the stack. A549 cells used in the CiGiP model showed increased levels of HIF1-α, decreased proliferation and reduced sensitivity to ionizing radiation. Authors identified three isogenic variants of A549 cells based on their metabolic radiosensitivity, which are known to differ in migration and proliferation in vivo [90].

Taken together, abovementioned studies have confirmed that 3D scaffold models can accommodate the TME oxygen content in a gradient-dependent manner for better therapeutic approaches.

## 10. Microfluidic Devices

Microfluidic technology provides exquisite control of any physical and chemical parameter of the cell culture in the device at the micrometer scale. This technology combined with 3D cell cultures has the potential to better replicate in vivo responses, as it effectively reproduces cell–cell and cell–matrix interactions, diffusion gradients of drugs, nutrients, oxygen, pH, and dynamic changes in microenvironmental parameters such as stiffness [104]. Different platforms can control oxygen concentration in three ways: introducing oxygen scavenging chemicals, altering the diffusional distance of oxygen or incorporating relatively oxygen impermeable materials.

Lung cancer metastasis was studied in a multiorgan microfluidic device [105]. A549 cells cultured inside the microfluidic device formed cancer mass and showed the EMT features. These cells were further used to evaluate the potential to invade the distant organs (brain, bone, liver). The microfluidic system was also used to monitor tumor models during anticancer treatment under varying oxygen conditions. Higher uptake of doxorubicin under a cycling hypoxia profile than under either chronic hypoxia was observed in breast cancer cells.

The study of Ch. Chang et al. [91] highlights the use of a polydimethylsiloxane–polycarbonate (PDMS–PC) hybrid microfluidic device for A549 cell culture under combinations of chemical and oxygen gradients. The drug testing results showed an increase in A549 cell apoptosis due to the hypoxia-activated cytotoxicity of tirapazamine. In addition, it was confirmed that the oxygen gradient plays an essential role during cell moving because the A549 cell migration assay demonstrated an aerotactic behavior. The authors claimed the device is promising to advance the control of in vitro microenvironments and allows to study of cellular responses under various physiological conditions simultaneously.

Another study harnessed the power of a 3D microfluidic chip to observe real-time changes in lung cancer cells after exposition to cobalt chloride (CoCl_2_), which simulates a hypoxic microenvironment [92]. It was demonstrated that Netrin-1 mediated EMT of A549 and PC9 cells in vitro, may be related to the phosphoinositide 3 kinase/AKT pathway, but only in a hypoxic microenvironment. The higher concentration of Netrin-1 was also found in NSCLC patients’ sera. Taken together, this finding obtained with the use of 3D microfluidic chip provided evidence that Netrin-1 promotes hypoxia-induced EMT in lung cancer cells and may be a potential therapeutic target.

An effective drug sensitivity test platform was designed by Z. Xu et al. [93] on microfluidic chip-based, 3D co-culture. A mixture of lung cancer and stromal cell lines, and cells from fresh lung cancer tissues were cultured in 3D under conditions mimicking the TME in vivo. The cells were treated with a panel of anticancer drugs according to a gradient concentration generator inside the chips to screen the appropriate chemotherapy schemes. The authors assayed the sensitivity to different anti-cancer drugs in parallel and accurately determined the appropriate dose of single and combined-drug chemotherapy schemes for eight patients. The presented microfluidic chip-based 3D co-culture may be useful to screen the appropriate chemotherapy schemes to guide individualized treatment in lung cancer.

A novel multi-flow microfluidic (MFM) system for the separation of circulating tumor cells (CTCs) from six out of eight NSCLC patients with high purity was discovered by J. Zhou et al. [94]. This device was constructed and configured based on the phenomenal effect of size-dependent inertial migration and allowed separation of CTCs from patients’ blood.

All abovementioned studies significantly confirmed that microfluidic systems are a promising platform for the hypoxia TME investigation due to the precise oxygen control facilitated by their small size scales.

## 11. 3D Bioprinting

3D bioprinting is an advanced fabrication technology that is used for creating tissues of one or more cell types that can mimic the 3D geometry and structure of native tissues. Three-dimensional bioprinting has emerged as a promising method to create reproducible but complex biological constructs by printing cell-laden hydrogel matrix precursors or bio-inks.

In the previously described study of A. Mondal et al. [89], SA-GL hydrogel was used to print NSCLC patient-derived xenograft cells and lung CAFs co-cultures. Rheological optimization of SA-GL hydrogel enhanced printability and viability of NSCLC xenograft cells and CAF co-culture, which allowed the 3D co-culture spheroid formation within the printed scaffold. Therefore, this model can be used for conducting high-throughput drug screening and other pre-clinical applications [78].

Another tumor-like lung cancer model was successfully created by X. Wang et al. [95] to evaluate the feasibility of utilizing it in biomedical applications. Three-dimensional bioprinting was used to fabricate a cell-laden hydrogel grid scaffold structure, using gelatin–sodium alginate lung cancer cell A549/95-D suspension as the bio-ink. Cell viability after the printing process remained over 90%, showing that the temperature and pressure changes the cells encountered during the 3D printing process, caused no serious damage. Biological properties of the printed cells, cell invasion and migration capabilities were checked by scratch test. Additionally, *MMP2* and *MMP9* genes expression was assessed. Results showed that both properties were improved in 3D printed cells compared to 2D cultured cells. Although the gelatin–sodium alginate system could simulate the extracellular structure and environment to a certain extent, this model can be only cultured for up to 28 days, as later, the structure would become disintegrated.

In a very interesting study of R. Utama et al., the formation of matrix-embedded multicellular spheroids prepared in high-throughput (HTP) was described [96]. Authors developed an enabling technology consisting of a bespoke drop-on-demand 3D bioprinter capable of HTP printing of 96-well plates of spheroids. This bioprint gave a high cell number and high cell viability. The HTP bioprinting of embedded spheroids was derived from various cell types, also human NSCLC H460 cells. Three-dimensional printed matrix-embedded spheroids features were compared to manually prepared spheroids by different approaches. H&E staining was performed on spheroid cross sections and showed that the cell arrangements and populations were very similar in both 3D bioprinted and manual spheroids at both days 3 and 6. Spheroids stained with phalloidin for F-actin organization and SYTOX green for nuclei were used to explore the cell arrangement, with no significant difference found between 3D bioprinted and manual spheroids. To visualize the organization of the spheroids, immunostaining for the cell proliferation protein Ki67 and the nucleus staining was prepared. Proliferating cells were consistently found on the periphery of the spheroid during the entire 6 days of investigation in both types of spheroids. Additionally, the percentage of the apoptotic marker cleaved caspase-3-positive cells was 0.4 and 1.7% for the 3D bioprinted and manual spheroid, respectively. Further, the authors checked the presence of hypoxic cells in the 3D bioprinted spheroids by HIF1-α immunolabeling. The analysis via fluorescence-activated cell sorting (FACS) showed equivalent positive cell populations of 5% in both the 3D bioprinted and manual spheroids. These data confirmed that generated the 3D bioprinted spheroids carry the important hypoxia characteristic of a 3D spheroid model. Additionally, the opportunity of HTP drug screening was investigated on neuroblastoma spheroids, exposed to doxorubicin. It was shown that sensitivity to spheroid size, embedding and how spheroids conform to the embedding affect the response toward doxorubicin [96].

In the presented study, the authors demonstrated 3D bioprinted spheroids that possessed important in vivo tumor-like characteristics found in manually prepared spheroids. Moreover, it was confirmed that the 3D bioprinting may be a robust HTP platform to screen biological and therapeutic parameters in the future.

## 12. Fluorescence Imaging of 3D Lung Cancer Models

Standard O_2_ imaging techniques described previously in Section 4b, have been applied clinically for tissue voxels assessment, but they are not useful for individual cell monitoring. Fluorescence imaging is a noninvasive method, which is highly advantageous for the study of 3D dimensional systems. During the 3D model generation, there has constantly been a need to control the tumor parameters. Phenotypic characterization of tumor spheroids, ECM accumulation or hypoxia occurrence can be visualized by light-sheet fluorescence microscopy. Recently, the 3D-3 model, based on the alginate microencapsulation strategy, composed from NSCLC cells, CAF and monocytes, were monitored over time by fluorescence imaging [106]. It was demonstrated that the 3D-3-culture recreates an invasive and the immunosuppressive TME, with accumulation of cytokines/chemokines, ECM elements and matrix metalloproteinases, supporting cell migration and promoting cell–cell interactions within the alginate microcapsules. The effectiveness of chemotherapeutic treatment of 3D-3-culture were also visualized by immunofluorescence.

In another study, confocal fluorescence microscopy was used to monitoring lung cancer cells with CD44 expression and showed varying invasiveness into the 3D hydrogel [107]. The tested biomimetic 3D hydrogel platform enabled to quantitative analysis of cell invasion and viability at the individual cell level and was developed using automated data acquisition methods. Within this system more detailed analyses of cellular responses to drug treatments are possible, allowing for more effective drug screens.

The fluorescence imaging for detection of hypoxic cell was commonly applied. The HIF-binding sequences were used to transcriptionally control the expression of fluorescent proteins under hypoxia in order to detect hypoxic cells in human prostate [108] and breast cancer [109] models. The unique system was generated by I. Godet et al. to track the fate of hypoxic cells that undergo reoxygenation in the bloodstream and lung [109]. This system allowed to permanently mark cells when they become hypoxic by triggering a fluorescent switch from red (DsRed, red fluorescent protein) to green (GFP, green fluorescence protein). Additionally, the fluorescence imaging indicated that cells exposed to hypoxia migrated away from the core of the spheroid, where they become hypoxic, to the more oxygenated periphery of the spheroid. The ability of the system to fate-map hypoxic cells was tested in 2D and in 3D spheroids, as well as in orthotopic and mouse models of breast cancer. The developed fate-mapping hypoxia system can be applied to another types of cancer that are critically affected by hypoxia.

## 13. Conclusions

Studies on 2D lung cancer models have been undermined by the fact that they do not accurately recapitulate the heterogeneity and complexity of the TME. The advances in the development of 3D NSCLC models offered new possibilities for hypoxia studies. Overall, created 3D models have many advantages compared to 2D cultures, because they may consist of one or more cell types, mimic the 3D geometry and structure of the native tissue and give the opportunity for precise oxygen control. Although significant progress has been made, most 3D models recapitulate only some particular aspects of the TME, thus further research is needed. What is still challenging in models reflecting tumor hypoxia in vivo is the extension of culture duration and the ability to simultaneously study cellular responses under different physiological conditions. What is relevant, described in the current review, is that in vitro models mimicking tumor hypoxia offer the alternative for preclinical studies over classical in vivo animal models.

In conclusion, the NSCLC in vitro 3D models mimicking tumor hypoxia reported in this study promise to contribute to the understanding of the pathogenesis of lung cancer and identifying the potential therapeutic targets. Additionally, the 3D models can be used for conducting high-throughput drug screening and other pre-clinical applications, which can facilitate the promotion of personalized medicine.

## Figures and Tables

**Table 1:** Difference between two-dimensional (2D) and three-dimensional (3D) cell culture.

Feature	2D	3D
**Cell morphology**	Shape changed, flat	Real shape, aggregates
**Cell polarity**	Partial	Replicate
**Cell proliferation**	High proliferation	Replication of proliferation rate in vivo
**Cell differentiation**	Nonspontaneous	Spontaneous may occur via cellular interactions
**Cell stage**	The same for all cells in culture	Heterogenous cell cycle stage (proliferating, hypoxic, quiescent, necrotic)
**Cell interactions**	Limited	Replicate in vivo
**Stiffness**	High	Low
**Culture formation**	Quick	Slow
**Culture duration**	Long	Short
**Cell culture**	High reproductivity	Low reproductivity
**In vivo like**	No	Mimics in vivo tissue and interactions: cell to cell, cell to extracellular matrix (ECM), cell to growth factor
**Tumoral heterogeneity**	Basic	Approximation to in vivo
**Exposure to nutrients, oxygen, drugs**	Equally	Variable access
**Drug response**	Rather lack of correlation with human tumors	Similar response pattern as in human tumors
**Costs**	Average	More expensive

**Table 2:** Various types of 3D NSCLC in vitro models (spheroids, organoids, 3D scaffolds, hydrogels, microfluidics, 3D bioprinting) that have been employed to generate hypoxic microenvironment in tumor.

Model	Cells	Results	Reference
Spheroids			
3D high-throughput screening system	A549	Hypoxia level of A549 spheroid was declined with TGF-β2 and elevated with TGF-β receptor I inhibitor (SB431542).	[78]
Spherical microtissues (hanging drop technology)	A549, Colo699 in combination with a fibroblast cell line (SV80) and two endothelial cell lines	Hypoxia marker (CA IX) was significantly expressed in microtissues that consisted of A549 cancer cells co-cultured with fibroblasts or endothelial cells.	[79]
3D cell co-culture collagen gel model	Human lung: adenocarcinoma cells (HCC), fibroblast cells (MRC-5) and macrophages	Hypoxia and/or serum starvation conditions induced elevated secretion of VEGF in the 3D co-culture model in vitro, but not MMP-1.	[80]
3D patient-derived tumor spheroids (PDS)	I/II stage NSCLC tumors	Long term 3D in vitro NSCLC model is useful for drug screening.	[81]
**Organoids**			
Primary lung cancer organoids	Primary lung cancer tissues and paired non-neoplastic airway tissues (epithelial cells)	Cultured for long-term expansion over 6 months without any change in spherical organoid morphology and maintained proliferation capacity.	[82]
Hybrid cancer organoids	Podoplanin-positive cancer-associated fibroblasts (CAFs) and NSCLC PC-9 cells	The proliferation of PC-9 cells in hybrid cancer organoids containing podoplanin-overexpressing CAFs was significantly higher.	[83]
**3D scaffolds**			
Decellularized scaffolds	HCC827, A549	Quantitative read-outs for proliferation, apoptosis and invasion were established in the complex 3D tumor model.	[84]
Microphysiologic 3D lung model (SISmuc platform)	A549	Antitumor activity of ROR1-CAR T was specific and potent against A549 lung cancer.	[85]
3D on human embryonic stem cell-qualified Matrigel-coated plates	Resections derived from NSCLC patients	3D system allowed for the isolation and expansion of stromal progenitors from tumor resections.	[86]
Synthetic scaffolds on porous PLGA	A549	Microparticles were used for A549 lung cancer cell culture.	[87]
Variotis tissue scaffold	NCI-H460	NSCLC cells showed enhanced expression of CA IX hypoxia marker.	[88]
**Hydrogels**			
Sodium alginate -gelatin (SA-GL)	NSCLC patient xenograft cells and lung CAFs co-cultures.	SA-GL hydrogel enhances printability and viability of NSCLC cells and CAF co-culture which allows 3D co-culture spheroid formation within the printed scaffold.	[89]
3D tissue-like constructCells-in-Gels-in-Paper (CiGiP)	A549	A549 cells showed increased levels of HIF1-α, decreased proliferation and reduced sensitivity to ionizing radiation.	[90]
**Microfluidic devices**			
PDMS–PC hybrid microfluidic device	A549	Drug testing results showed an increase in A549 cell apoptosis due to the hypoxia-activated cytotoxicity of tirapazamine.	[91]
3D microfluidic chip	A549 and PC9 cells in vitro	Netrin-1 mediated epithelial–mesenchymal transition (EMT) of A549 and PC9 cells in vitro was associated with the phosphoinositide 3 kinase/AKT pathway, but only in hypoxia.	[92]
Spheroids in device-assisted culture	Primary lung cancer cells, SPCA-1	Developed a high-throughput model for assessing drug sensitivities in vitro. There was a large discrepancy between drug sensitivity levels in 2D versus 3D.	[93]
Multi-flow microfluidic (MFM) system	Blood derived from NSCLC patients; NSCLC cell lines: HCC827, H460	Effective separation of circulating tumor cells (CTCs) from 6 out of 8 NSCLC patients.	[94]
**3D bioprinting**			
3D bioprinting using gelatin–sodium alginate-lung cancer cells suspension as the bio-ink	A549 and 95-D	Cell viability remained over 90%. Cell invasion and migration capabilities were improved in 3D printed cells compared to 2D cultured cells.	[95]
3D bioprinter for high-throughput printing of spheroids	NSCLC (H460), neuroblastoma (SK-N-BE(2), glioblastoma (U87vIII) cells	Organization of the printed spheroids, presence of apoptotic and hypoxic cells was comparable to manually prepared spheroids.	[96]

## Data Availability

No datasets were generated during the current study. Publicly available datasets were analyzed in this study. This data can be found here: String v11 database [https://string-db.org/]; Human Protein Atlas version 20.0 [http://www.proteinatlas.org]; HIF1A gene: [https://www.proteinatlas.org/ENSG00000100644-HIF1A/pathology/lung+cancer]; HIF2A gene [https://www.proteinatlas.org/ENSG00000116016-EPAS1/pathology/lung+cancer].
